# Temporal Effect of Adrenocorticotrophic Hormone on Adrenal Glucocorticoid Steroidogenesis: Involvement of the Transducer of Regulated Cyclic AMP-Response Element-Binding Protein Activity

**DOI:** 10.1111/j.1365-2826.2010.02096.x

**Published:** 2011-02

**Authors:** F Spiga, Y Liu, G Aguilera, S L Lightman

**Affiliations:** * Henry Wellcome Laboratories for Integrative Neuroscience and Endocrinology, University of BristolBristol, UK; † Eunice Kennedy Shriver National Institute of Child Health and Human DevelopmentNIH, Bethesda, MD, USA

**Keywords:** adrenal glands, steroidogenesis, ACTH, transducer of regulated CREB activity 2 (TORC2), salt-inducible kinase 1 (SIK1)

## Abstract

The availability of active steroidogenic acute regulatory protein (StAR) and side-chain cleavage cytochrome P450 (P450scc) are rate-limiting steps for steroidogenesis. Transcription of StAR and P450scc genes depends on cyclic AMP-response element-binding protein (CREB) phosphorylation and CREB co-activator, transducer of regulated CREB activity (TORC), which is regulated by salt-inducible kinase 1 (SIK1). In the present study, we investigated the relationship between TORC activation and adrenocorticotrophic hormone (ACTH)-induced steroidogenesis *in vivo*, by examining the time-course of the effect of ACTH injection (4 ng, i.v.) on the transcriptional activity of StAR and P450scc genes and the nuclear accumulation of transducer of regulated CREB activity 2 (TORC2) in rat adrenal cortex. ACTH produced rapid and transient increases in plasma corticosterone, with maximal responses between 5 and 15 min, and a decrease to almost basal values at 30 min. StAR and P450scc hnRNA levels increased 15 min following ACTH and decreased toward basal values at 30 min. Concomitant with an increase in nuclear phospho-CREB, ACTH injection induced nuclear accumulation of TORC2, with maximal levels at 5 min and a return to basal values by 30 min. The decline of nuclear TORC2 was paralleled by increases in SIK1 hnRNA and mRNA 15 and 30 min after injection, respectively. The early rises in plasma corticosterone preceding StAR and P450scc gene transcription suggest that post-transcriptional and post-translational changes in StAR protein mediate the early steroidogenic responses. Furthermore, the direct temporal relationship between nuclear accumulation of TORC2 and the increase in transcription of steroidogenic proteins, implicates TORC2 in the physiological regulation of steroidogenesis in the adrenal cortex. The delayed induction of SIK1 suggests a role for SIK1 in the declining phase of steroidogenesis.

The hypothalamic-pituitary-adrenal (HPA) axis is a neuroendocrine system that shows diurnal variation and is activated by stress to maintain homeostasis. Its activity is regulated by a feedforward mechanism involving the release of neuropeptides (corticotrophin-releasing hormone and vasopressin) from the paraventricular nucleus of the hypothalamus, which in turn stimulates anterior pituitary corticotrophs to secrete adrenocorticotrophic hormone (ACTH). ACTH subsequently induces glucocorticoid synthesis in the zona fasciculata of the adrenal cortex. Newly synthesised glucocorticoids are then released into the circulation to regulate a broad range of physiological processes, including metabolic and cardiovascular functions, immune response and behaviour.

Adrenal steroidogenesis is activated by the binding of ACTH to the specific melanocortin type-2 receptor (MC2R), cell surface seven-transmembrane domain G-protein-coupled receptor. Upon ACTH binding, MC2R undergoes conformational changes that activate adenylyl cyclase, leading to an increase in intracellular levels of cyclic AMP (cAMP) and subsequent activation of protein kinase A (PKA). Activation of PKA results in phosphorylation of cAMP-response element-binding protein (CREB), which binds to cAMP response elements (CRE) and induces gene transcription and synthesis of steroidogenic proteins.

Two proteins are of crucial importance in the acute induction of steroidogenesis from the precursor, cholesterol: (i) steroidogenic acute regulatory protein (StAR), which regulates the mobilisation of cholesterol and its transfer from the outer to the inner mitochondrial membrane (1, 2), and (ii) cholesterol side-chain cleavage cytochrome P450 (P450scc), which catalyses the cleavage of the cholesterol side chain to produce pregnenolone in the inner mitochondrial membrane (3). In contrast to peptide hormones, which, in basal conditions, are stored within the cytoplasm and released upon activation, glucocorticoids are synthesised *de novo* following ACTH-mediated stimulation of the adrenal. As glucocorticoids are released in a pulsatile manner with an hourly rhythm (4), glucocorticoid synthesis must be rapid and highly dynamic. Although it is clear that this pulsatility is important for glucocorticoid signalling (5, 6), surprisingly little is known about the mechanism underlying the pulsatile synthesis of glucocorticoids.

Transcriptional regulation of several CREB-inducible genes requires the activation by dephosphorylation and nuclear translocation of the CREB co-activator transducer of regulated CREB activity (TORC, also known as CRTC) (7–10). In basal conditions, transducer of regulated CREB activity 2 (TORC2) is sequestered in the cytoplasm in an inactive phosphorylated state by salt-inducible kinase 1 (SIK1) and other AMP-activated protein kinases (10, 11). It has been shown that inactivation of SIK1 by PKA allows dephosphorylation and nuclear translocation of TORC and consequent CREB dependent transcriptional activation. The TORC/SIK system has been implicated in adrenal regulation (12). SIK1 is present in the adrenals and studies in cell lines have shown that its inhibition by the protein kinase inhibitor staurosporin induces StAR and steroidogenic enzymes (13). The finding that SIK1 is responsible for TORC phosphorylation suggests that TORC is involved in the transcriptional regulation of the steroidogenic proteins (13).

In the present study, we have investigated the dynamics of glucocorticoid synthesis induced by a pulse of ACTH *in vivo* by measuring the time-dependent changes in transcription of StAR and P450 genes. Furthermore, in light of the importance of TORC2 and SIK1 in the regulation of steroidogenesis, the effect of ACTH was investigated on nuclear accumulation of TORC2 and on the transcription of the SIK1 gene.

## Materials and methods

### Animals

All experiments were conducted on adult male Sprague-Dawley rats (Harlan, Oxon, UK) weighing 250–300 g at the time of surgery. Animals were grouped housed four in each cage and allowed to acclimatise to the housing facility for a minimum of 1 week before the start of the experiment. Rats were maintained under standard environmental conditions (21 ± 1 °C) under a 14 : 10 h light/dark cycle (lights on 05.15 h) and food and water were provided *ad lib*. throughout the experiment. All animal procedures were approved by the University of Bristol Ethical Review Group and were conducted in accordance with Home Office guidelines and the UK Animals (Scientific Procedures) Act, 1986.

### Surgery

Animals were anaesthetised with a combination of Hypnorm (0.32 mg/kg fentanyl citrate and 10 mg/kg fluanisone, i.m.; Janssen Pharmaceuticals, Oxford, UK) and diazepam (2.6 mg/kg i.p.; Phoenix Pharmaceuticals, Gloucester, UK). Intravenous cannulation of the jugular vein was performed as previously described (14). The right jugular vein was exposed and a silastic-tipped (Merck Whitehouse, NJ, USA) polythene cannula (Portex, Hythe, UK), pre-filled with pyrogen-free heparinised (10 IU/ml) isotonic saline, was inserted into the vessel for ACTH administration. The free end of the cannula was exteriorised through a scalp incision and then tunnelled through a protective spring that was anchored to the parietal bones using two stainless steel screws and self-curing dental acrylic. The end of the protective spring was attached to a mechanical swivel that rotated through 360° in a horizontal plane and 180° through a vertical plane, allowing the rats to maximise freedom of movement. Following recovery from the anaesthesia, animals were housed in individual cages and left to recover for 5 days before starting the experiments. The cannula was flushed daily with the heparinised saline to maintain patency.

## Experimental procedures

Experiments started on day 5 after surgery at 14.00 h. To suppress endogenous ACTH secretion, rats were treated with the synthetic glucocorticoid methylprednisolone sodium succinate (250 mg, i.v., Solu-Medrone; Pharmacia, Sandwich, UK). This dose has been shown to inhibit corticosterone secretion within 30 min from injection and its effects last for over 5 h (15). Two hours after methylprednisolone injection, rats were treated with synthetic ACTH (Synacthen, [ACTH-(1–24)] fragment, 4 ng/0.1 ml, i.v.; Alliance Pharmaceutical, Chippenham, UK). Rats were overdosed with 0.5 ml of sodium pentobarbital (Euthatal, 200 mg/ml; Merial, Harlow, UK) before ACTH injection (t_0_) or 5, 15, 30 and 60 min (t_5_–t_60_) after ACTH administration. Trunk blood was collected on ice into tubes containing 50 μl of ethylenediaminetetraacetic acid (0.5 m; pH 7.4) and 50 μl of Aprotinin (500 000 KIU/ml, Trasylol; Bayer, EDTA, Newbury, UK). Plasma was separated by centrifugation and then stored at −80 °C until processed for corticosterone and ACTH measurements. Adrenal glands were collected and quickly dissected free of fat and decapsulated to separate the outer capsule containing the zona glomerulosa and the inner zones comprising the zona fasciculata and zona reticularis of the cortex and the medulla. Individual inner zones were immediately frozen in dry ice until processing for isolation of RNA for a reverse transcription quantitative polymerase chain reaction (RT-qPCR) (left adrenal) and protein extraction for western blotting (right adrenal).

### Hormone measurements

Total plasma corticosterone was measured by radioimmunoassay (RIA) using a citrate buffer (pH 3.0) to denature the binding globulin as previously described. Antisera was kindly supplied by Professor Gabor Makara (Institute of Experimental Medicine, Budapest, Hungary) and [^125^I] corticosterone was purchased from Izotop (Budapest, Hungary). The intra- and inter-assay coefficients of variation of the corticosterone assay were 14.1% and 15.3%, respectively.

ACTH in plasma was measured using 100 μl of plasma and RIA kit reagents (DiaSorin, Stillwater, MN, USA) in accordance with the manufacturer's instructions. This assay was chosen for its ability to equally recognise ACTH 1–24 and 1–39. The intra- and inter-assay coefficients of variation of the ACTH assay were 2.2% and 7.8%, respectively.

### Analysis of adrenal glands

#### RNA isolation and RT-qPCR

Total RNA was extracted from the inner zone of individual adrenals using TRIzol reagent (Invitrogen, Hopkinton, MA, USA), followed by purification using RNeasy mini kit reagents and column DNase digestion (Qiagen, Valencia, CA, USA) to remove genomic DNA contamination. Complementary DNA was reverse transcribed from 0.7 to 1 μg of total RNA as previously described (16). Primary transcript and mRNA accumulation of StAR, P450scc and SIK1 genes were evaluated using primer sequences designed to amplify nascent RNA (hnRNA) and mature RNA (mRNA) respectively, as shown in Table 1. Power SYBR green PCR mix (Applied Biosystems, Foster City, CA, USA) was used for the amplification mixture with each primer at a final concentration of 200 nm and 1.5 μl of cDNA for a total reaction volume of 12.5 μl. PCR reactions were performed on spectrofluorometric thermal cycler7900 HT Fast Real-Time PCR System; Applied Biosystems) as described previously (16). Samples were amplified by an initial denaturation at 50 °C for 2 min, 95 °C for 10 min and then cycled (45 times) using 95 °C for 15 s and 60 °C for 1 min. StAR, P450scc and SIK1 hnRNA and mRNA levels were normalised to glyceraldehyde 3-phosphate dehydrogenase mRNA as determined in a separate real-time PCR reaction. The absence of RNA detection when the reverse transcription step was omitted indicated the lack of genomic DNA contamination in the RNA samples.

**Table 1:** Primers Sequence.

RNA	Target	Primer	Sequence (5′ to 3′)
hnRNA	StAR	Forward	GCAGCAGCAACTGCAGCACTAC
		Reverse	GTGCCCCCGGAGACTCACCT
	P450scc	Forward	TGTGTGTGTGACCCCAGGAGAC
		Reverse	CCCAGGTCCTGCTTGAGAGGCT
	SIK1	Forward	TGTCAAGGAATGAGCGAGTG
		Reverse	TGAACTCCGACATGATCACC
mRNA	StAR	Forward	CTGGCAGGCATGGCCACACA
		Reverse	GGCAGCCACCCCTTGAGGTC
	P450scc	Forward	TGCGAGGGTCCTAACCCGGA
		Reverse	ACCTTCCAGCAGGGGCACGA
	SIK1	Forward	CCTCAGCAGTCTGGAGGTTC
		Reverse	TAAGGGCTGAAGCGAACTGT
	GAPDH	Forward	CCATCACTGCCACCCAGAAGA
		Reverse	GACACATTGGGGGTAGGAACA

StAR, steroidogenic acute regulatory protein; P450scc, cytochrome P450 side-chain cleavage; SIK1, salt-inducible kinase 1; GAPDH, glyceraldehyde 3-phosphate dehydrogenase.

#### Western blotting

Nuclear extracts from the inner zone of individual adrenals were prepared using NE-PER nuclear and cytoplasmic Extraction Reagent (Pierce, Rockford, IL, USA) in accordance with the manufacturer's instructions. Protein concentration was quantified by spectrophotometry using the BCA protein assay (Pierce). For western blot analysis of TORC2, 15 μg of nuclear or cytoplasmic proteins were loaded and separated in a 6% Tris-glycine gel (Invitrogen) and gels were run until the 50 kDa marker (Prestained protein ladder; Fermentas, Inc., Glen Burnie, MD, USA) ran off the gel. A 10% gel was used for protein separation for phospho-CREB. Proteins were transferred to a polyvinyl difluoride membrane (GE Amersham Biosciences, Piscataway, NJ, USA), incubated with 5% nonfat milk in 1 × Tris-buffered saline plus 0.05% Tween 20 (TBST) for 1 h and incubated overnight at 4 °C with anti-TORC2 (Calbiochem/EDM Chemicals, Gibbstown, NJ, USA), at a dilution of 1 : 6000, or anti-phospho-CREB (Ser133) (clone 10E9; Millipore, Billerica, MA, USA), at a dilution of 0.5 μg/ml. After washing with TBST, the membranes were incubated with a horseradish peroxidase-conjugated donkey antirabbit IgG at a dilution of 1 : 10 000. Immunorective bands were visualised using ECL Plus TM reagents (GE Amersham Biosciences) followed by exposure to BioMax MR film (Eastman Kodak; Rochester, NY, USA). After film exposure, blots were stripped and assayed for histone deacetylase 1 (HDAC1) and β-actin as a loading control. The intensity of the phosho-CREB (43 kDa), TORC2 (85 kDa), P-TORC2 (100 kDa), HDAC1 (62 kD) and β-actin (42 kDa) bands integrated with the area was quantified using a computer image analysis system, Image J (developed at the National Institutes of Health and freely available at: http://rsb.info.nih.gov). Data points for each gene were then normalised relative to the HDAC1 band in the respective sample. For each blot, samples from rats treated with ACTH (time points 5–60 min) were analysed as fold-inductions relative to time 0.

### Statistical analysis

Statistical significance of the differences between groups was calculated by one-way anova followed by Fisher protected least significant difference post-hoc test when appropriate. P≤0.05 was considered statistically significant. Data are represented as the mean ± SEM fold induction relative to t_0_ from the values in the number of observations indicated as appropriate.

## Results

### Effect of ACTH injection on plasma levels of ACTH and corticosterone

**Fig. 1:**
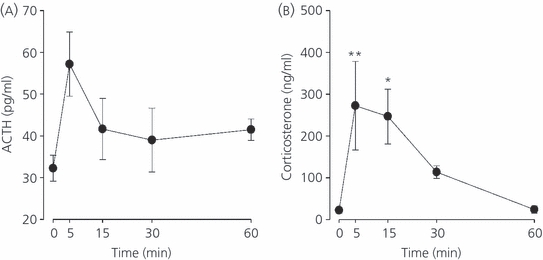
Time course of the effect of adrenocorticotrophic hormone (ACTH) on plasma levels of ACTH and corticosterone. ACTH (a) and corticosterone (b) levels are low in rats treated with methylprednisolone (250 μg/rat, i.v.) 2 h before ACTH injection (t_0_). Intravenous injection of synthetic ACTH (4 ng/rat, i.v.) results in brief elevation of circulating ACTH, which leads to an increase in corticosterone levels, with a significant increase 5 and 15 min after the injection. Data points are the mean ± SEM of the value obtained from three or four rats per group. *P < 0.01; **P < 0.005 compared to t_0._

The time-course of effect of a single injection of ACTH (4 ng) on plasma ACTH and corticosterone is shown in Fig. 1. ACTH and corticosterone levels in rats treated with methylprednisolone were low before ACTH injection. A small, but not significant, effect of the ACTH treatment on plasma levels of ACTH was found (F_4,20_ = 2.327; P = 0.101; Fig. 1a). By contrast, ACTH injection induced an increase in corticosterone levels (F_4,20_ = 5.024; P = 0.008; Fig. 1b) and this effect was significant 5 min (P = 0.005) and 15 min (P = 0.01) after injection.

### Effect of acute ACTH on StAR and P450scc gene transcription and mRNA accumulation

**Fig. 2:**
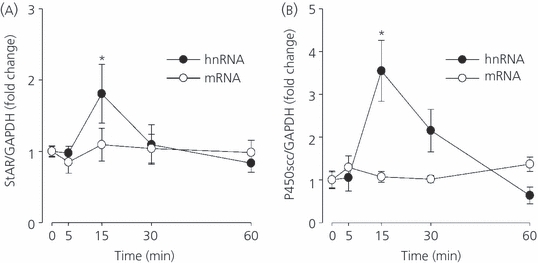
Time course of the effect of adrenocorticotrophic hormone (ACTH) on steroidogenic acute regulatory protein (StAR) and side-chain cleavage cytochrome P450 (P450scc) hnRNA and mRNA levels in the rat adrenal. Data points are the mean ± SEM of the values obtained from three or four rats per group expressed as the fold-change compared to values before ACTH injection (t_0_). Primary transcript (hnRNA) of StAR (a) and P450scc (b) was elevated 15 min after ACTH injection. No effect of ACTH on StAR and P450scc mature RNA (mRNA) was observed. *P < 0.05; **P < 0.0001 compared to t_0_.

The time-course of the effect of a single ACTH injection (4 ng) on the levels of primary transcript (hnRNA) and mRNA levels of the rate limiting steroidogenic proteins StAR and P450scc is shown in Fig. 2. A single injection of ACTH (4 ng) increased the levels of StAR hnRNA (F_4,21_ = 3.007; P = 0.048) and this effect was significant 15 min after ACTH injection (P = 0.016). This small dose of ACTH had no significant effect on the accumulation of StAR mRNA during the time period studied (F_4,21_ = 0.263; P = 0.898).

There was also an increase in P450scc hnRNA levels (F_4,21_ = 8.656; P = 0.00053) and this effect was significant 15 min after injection (P = 0.00027). Moreover, there was a trend towards significance 30 min after ACTH injection (P = 0.055). As observed for StAR protein, ACTH had no effect on the accumulation of P450scc mRNA (F_4,21_ = 0.874; P = 0.5).

### Effect of acute ACTH on nuclear phospho-CREB and TORC2

**Fig. 3:**
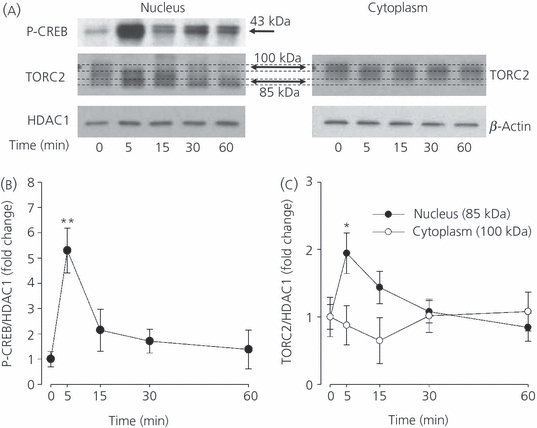
Time course of the effect of adrenocorticotrophic hormone (ACTH) on nuclear levels of phospho-cyclic AMP-response element-binding protein (P-CREB) and nuclear and cytoplasmic levels of transducer of regulated CREB activity 2 (TORC2) in the rat adrenal. Data points are the mean ± SEM of the values obtained from three or four rats per group expressed as the fold-change compared to values before ACTH injection (t_0_). Gels for nuclear and cytoplasmic proteins were run simultaneously in the same electrophoresis apparatus. The dotted lines in (a) show the alignment of the bands in nuclear and cytoplasmic proteins according to the molecular size markers indicating the shift in migration of the bands. Nuclear levels of P-CREB were elevated 5 min after ACTH injection (a, b). Nuclear levels of nonphosphorylated TORC2 (85-kDa band) were elevated 5 min after ACTH injection, whereas no effect of ACTH was observed on phosphorylated TORC2 (100-kDa band) in the cytoplasm (a, c). *P < 0.05; **P < 0.001 compared to t_0_. HDAC1, histone deacetylase 1.

The ability of a small ACTH dose to induce phosphorylation of CREB and activation of the CREB co-activator, TORC 2, in the rat adrenals was investigated using western blotting (Fig. 3). The time-course of effect of ACTH injection on nuclear P-CREB is shown in Fig. 3(a,b). There was a significant effect of ACTH treatment on nuclear levels of P-CREB (F_4,16_ = 6.571; P = 0.0048). This effect was transient, with a marked increase in nuclear P-CREB 5 min after ACTH injection (P = 0.001), and returned to levels not significantly different from basal by 15 min.

The time course of the effect of ACTH on nuclear accumulation of TORC2 in the rat adrenals is shown in Fig. 3(a,c). Western blot analysis of TORC2 in adrenal nuclear and cytoplasmic proteins revealed bands from approximately 85 to 100 kDa corresponding to the molecular size of dephosphorylated and phosphorylated forms of TORC2, respectively (Fig. 3a). In the cytoplasm, there was a predominant band of approximately 100 kDa (Fig. 3a). In the nucleus, the 100-kDa band was very low, although there was an increase of lower molecular size bands of approximately 85 and 95 kDa. Semiquantitative analysis of the 85-kDa band (nonphosphorylated TORC 2) revealed a time dependent increase (F_4,16_ = 3.315; P = 0.048; Fig. 3c). This increase of nuclear TORC 2 was statistically significant 5 min after ACTH injection (P = 0.02) and returned to levels not significantly different from basal by 15 min. By contrast, analysis of the 100-kDa band (phosphorylated TORC2) revealed no significant effect of ACTH treatment on cytoplasmic P-TORC2 (F_4,16_ = 0.342; P = 0.844).

### Effect of ACTH on SIK1 gene transcription and mRNA accumulation

**Fig. 4:**
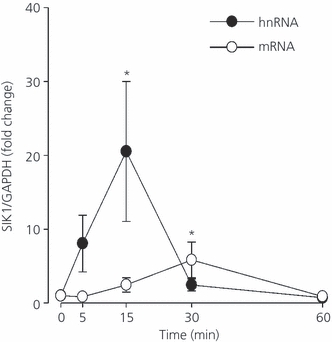
Time course of the effect of adrenocorticotrophic hormone (ACTH) on salt-inducible kinase 1 (SIK1) hnRNA and mRNA levels in the rat adrenal. Data points are the mean ± SEM of the values obtained from three or four rats per group expressed as the fold-change compared to values before ACTH injection (t_0_). Primary transcript and mature RNA (mRNA) of SIK1 were elevated 15 and 30 min, respectively, after ACTH injection. *P < 0.005 compared to t_0_. GAPDH, glyceraldehyde 3-phosphate dehydrogenase.

The time-course of effect of ACTH injection on the TORC2 suppressor kinase, SIK1 hnRNA levels is shown in Fig. 4. ACTH treatment increased the levels of SIK1 primary transcript (hnRNA) (F_4,21_ = 3.945; P = 0.019) and this effect was significant 15 min after ACTH treatment (P = 0.004). There was also an effect on mature SIK1 RNA (mRNA) (F_4,21_ = 3.735; P = 0.023) and this effect was significant 30 min after ACTH injection (P = 0.005).

## Discussion

Glucocorticoid secretion in rats exhibits an ultradian rhythm characterised by release of pulses of corticosterone from the adrenal gland at a rate of one pulse per hour. Although increasing evidence emphasise the importance of glucocorticoid pulsatility for HPA responsiveness to stress (17) and for glucocorticoid receptor (GR)-mediated gene transcription (5, 6), the dynamics of pulsatile synthesis and secretion of corticosterone have not been studied. The present study aimed to investigate the rapid effects (5–60 min) of low doses of ACTH, mimicking ultradian pulses on adrenocortical function. Furthermore, the use of intronic qRT-PCR to determine changes in primary transcript allowed us to estimate rapid changes in the transcriptional activity of genes involved in steroidogenesis. The data obtaned clearly show that minor increases in plasma ACTH induced rapid and transient increases of plasma corticosterone resembling an ultradian pulse. This was paralleled by sequential phosphorylation of CREB and nuclear translocation of TORC2, increases of StAR and P450scc transcription, and an increase of transcription and mRNA accumulation of the TORC2 regulatory kinase, SIK1.

Previous investigations *in vivo* examining the long-term effects of high ACTH doses have demonstrated the ability of ACTH to induce the steroidogenic proteins StAR and P450scc (18, 19). In the present study, the injection of a very small ACTH dose was sufficient to induce rapid increases in plasma corticosterone with characteristics both of amplitude and duration comparable to an endogenous corticosterone pulse (14). This effect of ACTH was evident despite the barely detectable increases in plasma ACTH. Although it is possible that plasma levels reached higher levels before the first time point measured (5 min), the data indicate that adrenal fasciculata cells are distinctively sensitive to small elevations in circulating ACTH.

The rapidity of this response is consistent with *in vitro* studies demonstrating that ACTH can initiate corticosterone synthesis within approximately 3 min (20–22). The time course of ACTH induced changes in corticosterone levels and in transcription of the steroidogenic enzymes was quite remarkable. Although corticosterone rapidly reached peak levels by 5 min, StAR and P450scc hnRNA had not changed by this time and only peaked at 15 min. By contrast to the lack of change in StAR or P450scc mRNA within 60 min of injection of 4 ng ACTH in the present study, a previous report by LeHoux *et al*. (18) described a delayed increase in StAR mRNA after 30 min of a larger dose of ACTH. Thus, it is likely that smaller increases in mRNA synthesis in the present study were masked by rapid mRNA turnover as a result of translation of pre-existing mRNA. On the other hand, the measurement of hnRNA in the present study revealed increases in StAR and P450scc transcription after these minor increases in circulating ACTH. Although rapid, it is also clear that transcriptional activation does not precede corticosterone synthesis and secretion. What is evident from both studies is that newly synthesised and released corticosterone following ACTH injection must depend upon ACTH-induced post-transcriptional (likely translational and post-translational) changes of steroidogenic proteins rather than the transcriptional response. Thus, newly transcribed hnRNA would serve to restore steady-state mRNA pools. In this regard, ACTH has been shown to modulate post-translational modification of StAR protein via proteolytic processing (23) and phosphorylation (24).

Another remarkable finding was that nuclear levels of phospho CREB and its co-activator, TORC2, increased within 5 min of the injection of ACTH, well before the change in StAR and P450scc transcription, and was already declining by 15 min. The importance of TORC2 translocation to the nucleus in steroidogenesis has been previously suggested by *in vitro* studies in cell lines demonstrating that the kinase inhibitor, staurosporin (known to inhibit SIK1 and induce TORC2 translocation to the nucleus) up-regulates the expression of steroidogenic proteins (13). The present study provides the first demonstration that minor rises in circulating ACTH, capable of mediating ultradian pulses of glucocorticoids, cause rapid increases in the 85-kDa band corresponding to dephosphorylated TORC2 in the nucleus, which precedes transcriptional activation of StAR and P450scc. Although the western blot clearly shows the more rapid migration of the nuclear bands compared with the highly phosphorylated cytoplasmic bands (Fig. 3a), slower migration bands were also present in the nuclear fractions. The 100-kDa band, which was very weak and did no change after ACTH, may correspond to cytoplasmic protein contamination in the nuclear fractions. The identity of the 95-kDa band is less clear and it is likely to correspond to partially phosphorylated forms. In this regard, it was recently reported that, in addition to Ser 171, SIK1 phosphorylates TORC2 also at Ser 307, and phosphorylation at this site inhibits nuclear localisation of TORC2 (25). Therefore, it is possible that the p-TORC2 band observed in the present study represents phospho-Ser171/dephospho-Ser307-nuclear-TORC2.

Nuclear translocation of TORC2 is initiated by PKA-dependent phosphorylation of SIK1 at Ser577, leading to its inactivation (26). SIK1 is responsible for maintaining the TORC2 phosphorylation at Ser^171^ and Ser^275^. In its phosphorylated form, TORC2 is transcriptionally inactive and remains in the cytoplasm bound to the scaffolding protein 14-3-3. Thus, ACTH-mediated activation of PKA would initiate StAR and P450scc transcription through CREB phosphorylation and simultaneous inactivation of SIK1, inhibiting TORC2 phosphorylation and allowing its translocation to the nucleus.

Interestingly, consistent with studies demonstrating the rapid induction of SIK1 by cAMP in the steroidogenic cell line Y1 (27) and rat pinealocytes (28), ACTH also induced SIK1 transcription and mRNA. Although mRNA levels do not directly relate with the levels of SIK1 protein or its inactive/active state by phosphorylation/dephosphorylation, the dynamic changes in expression induced by the ACTH injection supports the proposal that SIK1 plays an important role in the control of steroidogenesis.

The evidence suggesting that ACTH is able to both inactivate (via cAMP-PKA mediated phosphorylation) and induce SIK1 raises the exciting possibility that ACTH mediated inactivation and activation of SIK1 could be part of an intracellular feedback mechanism responsible for pulse generation in the adrenal cortex.

In summary, the data obtained in the present study clearly demonstrate that small elevations of circulating ACTH by a single i.v. injection in methylprednisolone-suppressed rats induce a pulse of corticosterone of amplitude and duration similar to physiological ultradian peaks. The parallel changes in StAR and P450scc transcription suggest that pulsatility involves the dynamic regulation of steroidogenic proteins. Although the signalling pathways leading to SIK1 activation and the role of SIK1 on the termination of transcription are currently under investigation, it is tempting to speculate that, concomitant with phosphorylating CREB, ACTH-mediated PKA activation will initially inhibit SIK1 by phosphorylation and then induce it, resulting in a biphasic change in SIK1 activity. This process would result in a pulse of CREB-activated gene transcription of both StAR and P450scc hnRNA.
